# Voices From Diversity, Equity, and Inclusion Leaders in Emergency Medicine, Understanding Their Experiences

**DOI:** 10.1111/acem.70322

**Published:** 2026-05-14

**Authors:** Ryan E. Tsuchida, Rebecca J. Schwei, Dowin Boatright, Aasim I. Padela

**Affiliations:** ^1^ BerbeeWalsh Department of Emergency Medicine, School of Medicine and Public Health University of Wisconsin Madison Wisconsin USA; ^2^ Department of Emergency Medicine New York University Grossman School of Medicine New York New York USA; ^3^ Department of Emergency Medicine Medical College of Wisconsin Milwaukee Wisconsin USA; ^4^ Center for Bioethics and the Medical Humanities, Institute for Health and Equity Medical College of Wisconsin Milwaukee Wisconsin USA

**Keywords:** administration, faculty development, leadership, racism, workplace equity

## Abstract

**Background:**

While EM departments have designated new leadership roles to focus on diversity, equity, and inclusion (DEI) in recent years, the experiences of DEI leaders have not been thoroughly investigated. The purpose of this study was to describe the factors that impact the effectiveness of DEI leaders within EM.

**Methods:**

We conducted a national qualitative study of DEI leaders in EM. Participants participated in a 60‐min semi‐structured interview. An interview guide was pilot‐tested and iteratively refined. The interview audio was recorded and professionally transcribed. Two team members developed a codebook, independently coded transcripts, and generated categories using content analysis. We analyzed the interview transcripts using inductive and deductive content analysis. Inductive analysis allowed us to identify emerging categories, while deductive analysis allowed us to overlay Camara Jones' Allegory of the Levels of Racism to our data.

**Results:**

We completed 24 interviews, representing 21 unique institutions in 14 states. In our adapted allegory, the DEI leader is represented centrally within the ecosystem. The effectiveness of the DEI leader is influenced by institutionally mediated, personally mediated, and internally mediated factors. Institutionally mediated factors included the macroenvironment, administrative positioning, and promotion pathways. Personally mediated factors included communication skills, ongoing leadership development, change management prowess, and seeking colleagues' support. Internally mediated factors included personal commitment to DEI work, as well as feelings of workplace inclusion, imposter syndrome, and tokenism.

**Conclusions:**

This qualitative analysis of EM DEI leaders highlights how factors at various levels influence their experience. Jones's allegory helps conceptualize how a DEI leader functions in a dynamic, continually evolving environment that is sometimes beyond the leader's control. Our research identifies opportunities at the personal, departmental, and institutional levels, such as maintaining a personal commitment to the work, supporting leadership development, and improving administrative positioning that can assist DEI leaders' effectiveness.

## 1 Introduction

Students, faculty, and staff from disadvantaged and marginalized backgrounds lack representation in medicine [1, 2, 3], especially in leadership positions, and experience disproportionate rates of mistreatment and discrimination [4]. In response, academic institutions have established roles such as Deans for Diversity, Equity, and Inclusion (DEI) and Chief Diversity Officers to develop pipeline programs, combat workplace discrimination, and otherwise undertake initiatives that inculcate a workplace culture of tolerance and equity [5]. In parallel, health systems have implemented novel approaches for recruiting clinicians from backgrounds more representative of the patient populations they serve, with the goal of reducing health disparities [6, 7, 8]. Recent literature has highlighted four categories of challenges for institutional DEI leaders, including (1) broad scopes of work, unclear expectations, and limited resources, (2) insufficient institutional investment, (3) the lack of evidence‐based frameworks, theories of change, or standards of expertise, and (4) the negative personal toll of DEI work [9].

More recently, academic departments, including departments of emergency medicine, have created DEI leadership roles to similarly foster programs and initiatives aimed at recruiting trainees and faculty from diverse backgrounds, reducing health care disparities, and fostering an inclusive working environment at the department level [1, 10]. There has been scant research on how these roles in academic medical departments unfold. Our prior work found that DEI emergency medicine leaders had been doing DEI work for an average of 5 years prior to taking on an inaugural role [11]. In many cases, formal roles were newly created around 2020, and the mechanisms influencing their effectiveness have not been examined. Our survey found that people in such roles, such as a Vice Chair or Director for DEI, lacked significant resources, such as a budget or funded time effort [11], which may negatively influence their effectiveness. Only one available study has qualitatively described the experiences of DEI leaders in emergency medicine. Molina et al. [12] interviewed 21 DEI leaders who reported challenges related to nonlinear pathways toward promotion, undefined roles and expectations, and variable perceived value. We build upon that work in this qualitative study aimed at describing factors that influence the success of EM DEI leaders.

## 2 Methods

### 2.1 Recruitment and Sampling

We recruited a convenience sample of DEI leaders in emergency medicine from across the United States via targeted emails for virtual, semi‐structured interviews. Specifically, we utilized the Society for Academic Emergency Medicine's Academy for Diversity and Inclusion in Emergency Medicine (ADIEM) listserv. Additional recruitment was done by the first author, soliciting participation at ADIEM academy meetings. All interested participants were provided a link to schedule a virtual interview. Participants were eligible if they self‐identified as holding a DEI leadership position in emergency medicine in the United States, including physicians and PhD‐trained emergency medicine faculty. Individuals were excluded if their DEI leadership role was exclusively outside of their EM department. We continued conducting interviews until we reached data saturation, defined as the point at which prior data were repeated, and new data no longer contributed to addressing the research question, across the domains of inquiry: institutionalized, personally mediated, and internalized factors of effectiveness [13].

Participants provided verbal consent during the interviews, which took place from July 2024 through November 2024 on a secure online platform, and received a $50 Amazon digital gift card following the interview. The local institutional review board approved all study activities. We followed the consolidated criteria for reporting qualitative research guidelines [14]. The funding organization had no role in the conduct or reporting of the study.

### 2.2 Theoretical Framework

We used Camara Jones' Theoretical Framework of the Levels of Racism to structure our interview guide and organize high‐level domains of developed categories [15]. Jones uses the image of two flower boxes and a gardener to illustrate three levels of racism: institutionalized, personally mediated, and internalized. In her allegory, the soil represents the institutionalized racism, the gardener's disdain for the pink flowers that did not flourish and their continued efforts to hold the pink flowers back represent personally mediated racism and the internalized racism presents as the pink flowers telling the bee not to pollinate them because the red flowers are better. More specifically, institutionalized racism is defined as differential access to the goods, services, and opportunities of society by race. Personally mediated racism is defined as prejudice (i.e., differential assumptions about abilities, motives, and intentions) and discrimination (i.e., differential actions) towards others according to their race. Finally, internalized racism is defined as the acceptance by members of the stigmatized races of negative messages about their own abilities and intrinsic worth. In this framework, the gardener is the government, the group that has the power to decide, act and controls the resources.

### 2.3 Design and Procedure

We employed semi‐structured interviews to investigate the factors that influence the effectiveness of DEI leaders in emergency medicine. Questions were developed to investigate these factors and pilot‐tested with three DEI leaders in academic medicine (see Appendix S1). Transcripts of the pilot interviews were reviewed by study team experts (AIP and DB) and discussed collectively at team meetings to refine the interview guide and interviewer methods. Interview questions were primarily open‐ended to gather participants' immediate thoughts. Follow‐up probes were guided by the study team's knowledge of existing DEI and leadership literature, including Camara Jones' Theoretical Framework [9, 11, 15, 16, 17]. The guide was iteratively refined to tweak some stems or probe further into topics that took place during the interviews.

Interviews were conducted by RET in his work office, a male emergency medicine physician who previously served as the DEI lead in his department. RET had previously met and, in some cases, worked with interviewees as colleagues. Potential bias due to perceived power imbalances or pre‐existing relationships was managed by his adopting a non‐judgmental approach to the inquiry where RET affirmed interviewees' experiences without offering any value‐based judgments or feedback based on his own experiences or scholarly insight. Moreover, to mitigate against social desirability‐type biases, the interview questions covered both positive and negative elements of DEI work, and the interviewer focused on whichever elements were more salient to the participant. RET practices in a state with a mix of permissive and restrictive elements to DEI work, though he was not significantly personally impacted by anti‐DEI policies.

RET provided periodic debriefing and received feedback from DB and AIP, practicing emergency medicine physicians with advanced training in qualitative methods and expertise in DEI. After each interview, memos were handwritten, and questions were refined to encourage participants to elaborate on emerging categories. All interviews were conducted on Zoom, with both audio and video streaming, and lasted 45–60 minutes. The audio was recorded and transcribed verbatim by a professional transcription service. Due to the politicized environment surrounding DEI work and the risk of de‐identification with a small number of participants known to the intended readership audience, we did not systematically collect demographic information from participants beyond their region of the country.

### 2.4 Content Analysis

We analyzed the interview transcripts using both inductive and deductive content analysis [18, 19]. Data analysis consisted of several steps. Based on the initial impressions of the interviewers, RET created an initial codebook. Then, RET and RJS jointly reviewed three interviews to immerse themselves in the data. RJS, a female PhD‐trained researcher with advanced training in qualitative methods, participated in the analysis. After this preliminary coding, the analysts met and revised definitions, reorganized the coding schema, and added additional codes (see Appendix S1). For the remaining interviews, RJS conducted the coding while RET reviewed. RET and RJS regularly met to refine codes and definitions. Throughout the coding and analysis stage, ongoing team meetings with RET, RJS, DB, and AIP were held to resolve any coding and interpretative ambiguities. NVivo 15 was used to facilitate analysis.

Inductive coding was completed first and allowed us to identify emerging categories. Through iterative review and comparison of codes, we refined and organized these inductive categories. Once inductive coding was complete, we noticed that many of the categories mapped well to Camara Jones' Theoretical Framework of the Levels of Racism. We thus used deductive analysis to map our categories onto the framework. In some instances, we combined categories to make one overarching category; for example, we combined current events, federal or national politics, pushback and state politics to generate the category macro environment. In other situations, we reorganized the codes to situate one category within a larger category (i.e., minority tax as a subcategory of pathways for promotion). In this way, we developed an organizational framework to present the study results.

## 3 Results

### 3.1 Sample Description

**FIGURE 1:**
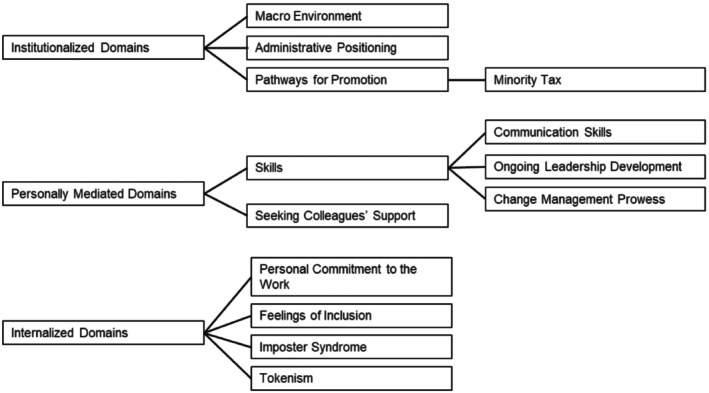
Map of the Results by Domain and Category. This figure highlights the institutionalized, personally mediated and internalized domains and categories and sub‐categories within each domain that impacted DEI leaders' effectiveness.

**FIGURE 2:**
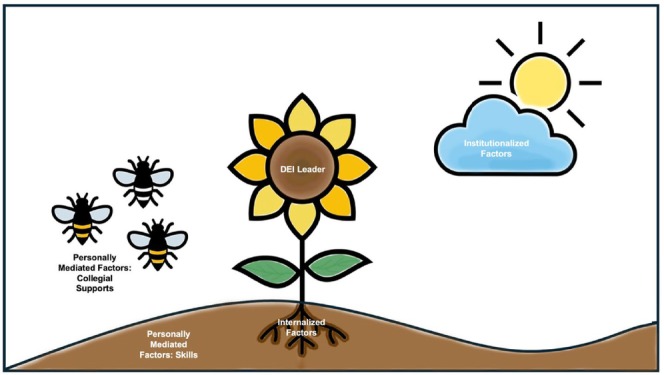
Nurturing Effective DEI Leaders. This image is an extension of Camara Jones' allegory, the Gardener's Tale. The DEI leader is the sunflower; the sun and cloud represent institutionalized factors that will influence the effectiveness of DEI leaders; the soil where the plant is planted and the bees represent personally mediated factors; and the characteristics of the roots represent internalized factors.

A total of 26 people expressed interest in being interviewed; however, two individuals did not show up at their pre‐scheduled interview time. The final sample consisted of 24 interviewees across 21 different healthcare systems, working in 14 states across the United States. In the next sections, we use three domains: institutionally, personally, and internally mediated, to organize several categories that interact and create the environments where DEI leaders work. Figure 1 is a map of the results by domain and category. Figure 2 is a visual representation of the expanded allegory of Jones' framework where the institutionalized domains are represented by the sun and cloud, the personally mediated domains are represented by the soil and bees, and the internalized domains are represented by the roots.

### 3.2 Institutionalized Domain (Sun and Cloud)

Categories within the institutionalized domain refer to the environment surrounding the DEI leader that impacts their effectiveness. This includes the macro environment, the administrative positioning of the role, the financial resources allocated to the work, and the pathways for promotion.

#### 3.2.1 Macro Environment

We defined the macro environment as the external context affecting the DEI leader and their work. This included both the state environment and the environment within an institution. One participant described the motivation behind DEI positions being created: “The climate that was in 2020 with the social justice issues, with George Floyd and things like that, I think that was when there was a big push to create this [DEI] committee.” (1011) Another participant echoed the climate saying:

With the social unrest that happened in 2020 and COVID, as you know, many departments started creating DEI leaders in varying positions to help them with their mission of advancing health equity, social justice kind of work. So then I was appointed to that position as the inaugural in our department. (1001)

However, over the past 5 years, the macro environment has shifted. One participant described the current macro environment this way: “Now there's apathy… we don't have to pay attention to [DEI] anymore. Let's move onto something else.” (1002).

For some states, animosity against DEI work was described as so intense that state‐level DEI laws were enacted to restrict the work. Participants explained how these laws often banned terms like ‘diversity.’ “We're stripped a lot of the programming inside of our university so that we step away from that concept of diversity, the naughty D‐word as I like to say.” (1003) Other participants described how state laws will ultimately lead to decreased scholarly work and production.

We have a law now that tenure has to be reviewed every five years by some sort of government‐appointed body. And if the work you're doing in tenure doesn't comport with whatever values they decide or of the day, then they can strip you of your tenure and fire you. So a lot of faculty in the DEI space are not going for tenure anymore. They're kind of decreasing their scholarly work and production. (1008)

Due to increased state legislation limiting DEI work, participants shared innovative solutions to address the changing laws, such as reframing efforts. “And so our work, while it's continued, it's almost like we've had to reframe the work, you know, just to suit those above us, I guess.” (1021) Other participants described ensuring the opportunities they offer are available to everyone. “The only major change has maybe been in the way that we talk about some of the opportunities that we offer… ensuring that there is equal opportunity for everybody to apply for programs that we offer and just changing some of the language.” (1005).

#### 3.2.2 Administrative Positioning

Administrative positioning described the DEI leader's role within their department, including specific leadership responsibilities, decision‐making authority, direct communication channels to the Chair, and access to financial resources. One participant emphasized the importance of having a designated DEI Vice Chair role. “Creating our Vice Chair of DEI position, creating things that actually come with money, with time, with an ear to the Chair and things like that. I appreciated that [the role] didn't just come with words, that there actually was action created and resources allocated.” (1019) Likewise, another participant mentioned how having a seat at the decision‐making table was vital for their effectiveness.

So I think that is really integral and kind of making sure the culture remains, that there is a level of sensitivity to certain areas and certain topics that if I wasn't at the table where those decisions were being made, I think I would have much less impact. So I think that level of opportunity and really, to be a part of those discussions is really, really important. (1018)

Having a substantial budget or buy‐down made participants feel valued for their work. One participant said, “My time is definitely bought down, and it's in line with other kind of like newer director roles…So I think that having that protected time is definitely a way that the department shows its intentionality and its commitment to making this a real thing.” (1021). Participants described how limited financial resources for DEI work constrained the work they could accomplish. “If you have a chair that's not going to give you any money to do DEI stuff and isn't going to do any buy‐down for anyone that's related to DEI, then you're going to struggle.” (1007).

#### 3.2.3 Pathways for Promotion

Pathways for promotion was the third institutionalized sub‐category and was defined as discussions surrounding whether DEI skills, experiences, and performance facilitated formal professional advancement. Participants described situations where DEI work was not currently considered relevant in academic promotion. “I used to think that [DEI work] helped a lot because we had a very specific section on DEI activities that you had to do something inside of that realm in order to be promoted in the future. Again, with our political landscape, that has been stripped.” (1003) Participants emphasized the importance of receiving academic credit for this work. One participant said, “This work has to be recognized for its merit. And it has to be taken into consideration under promotion.” (1014).

##### 3.2.3.1 Minority Tax

Minority tax, specifically, was cited as an important consideration within the broader category of pathways for promotion. Minority tax has been defined as the additional responsibilities and expectations placed on underrepresented minorities compared to those who identify with the dominant culture, while also coping with and managing daily, institutionalized bigotry in professional and personal lives [20]. Participants described prior experiences with minority tax.

I was just talking to a good friend of mine about this, …, wow, this seems like they're asking a lot of you. All of this stuff is taking a lot of time. Are you getting any time for this? Are you getting any sort of monetary support? …And we did start essentially having this discussion about minority taxes, okay, do you feel you're being looked at to do this stuff, not just because, okay, fine, you want to do it, but also because you are a minority who then is like, oh, okay, and you're interested in this work? (1004)

In contrast, participants noted that having a formal position with monetary compensation or protected time could help counteract the minority tax. “And so it was a relief, and I felt really good to be able to finally not be facing that minority tax in the same kind of way, by being able to do this work and being adequately compensated for it in whatever form that is in terms of money or time or whatever.” (1005) This quote illustrates how administrative positioning and compensation for DEI work intersect with feelings surrounding the minority tax.

### 3.3 Personally Mediated Domain (Soil and the Bees)

Categories within the personally mediated domain are those that influenced DEI leaders' effectiveness, including the soil where the plant grows (skills, soil) as well as the sunflower's relationships and personal support systems in other parts of their environment (seeking colleagues' supports, bees).

#### 3.3.1 Skills

Participants identified a range of skills essential for effective DEI leadership. These included communication skills, ongoing professional development, and change management prowess.

##### 3.3.1.1 Communication Skills

Communication skills, such as code switching, were defined as essential skills involved in communicating with and relating to other people. Participants explained that to be effective DEI leaders, they needed to adjust their default communication styles. One participant described this adjustment this way, “And there's a lot of similarities between… languages, but they're not the same. But having that extra degree and having that extra exposure allowed me to be bilingual. And so I can speak in public health terms, and I can speak in medical terms.” (1010) Participants acknowledged that effective communication and respectful interactions are crucial skills for successful DEI leadership.

But I think I'm a very direct communicator, tend to be a pretty like passionate person about things that matter to me. And, you know, it was, there was some growth there in learning that when you are direct and passionate, it doesn't always play very well in a room… and can certainly take on a negative tone or a perception of, you know, that you're attacking the person, the policy, the whatever. And really, recognizing that that's not the way that you can ever approach any of these conversations in particular because as soon as the person you're speaking to becomes defensive, like they've shut down. You've lost them, and you've lost the way to communicate. (1015)

##### 3.3.1.2 Ongoing Leadership Development

Ongoing leadership development was identified by DEI leaders as essential for effectiveness. One participant described previously participating in formal leadership training programs, which helped develop strong leadership skills. “I have been encouraged to do leadership workshops and classes and things like that. So I've done that. Part of my growth in this leadership role is, what other aspects does it take to develop a leader?” (1013) One participant described the value of intentional development of leadership skills in this way,

I think purposeful development in this area is really important… I think it's something you have to be very purposeful about. There are certainly natural leaders, but I think, particularly in academic medicine, particularly in this space, it helps to have training and support and a network to get to, to get you there completely. (1009)

##### 3.3.1.3 Change Management Prowess

Change management prowess referred to the ongoing, often slow and challenging journey of driving meaningful change related to DEI work. Participants described DEI work as being much longer and more complex than they initially expected. One participant said,

So what I was probably most unprepared for was the length of time that it takes to make change in DEI. You know, my thought was, how long could this take, six months, a year, a year and a half tops? … It took us about ten years to get to where I thought we should be in a year, year and a half. So I was most unprepared for the timeframe of change. (1002)

Participants described needing to take small steps and accept gradual change. “I think that with a small group, it's hard to make big, broad steps. You got to start with small steps.” (1016). One participant described how challenging it was to accept the slow pace of progress this way.

There have been times in my career I have been extremely frustrated with systems, and to the point where I've been ready to give up, leave academic medicine, leave my department, etc., because, you know, it's one of those things where you feel like you're just banging your head against a brick wall, and you're not making a dent. You are just, and you're only frustrating yourself because the system isn't changing… And my mentors had to tell me to stay the course, and I think that was the biggest piece of advice for me, what's the saying, the arc may be long, but it ultimately bends towards justice. (1010)

#### 3.3.2 Seeking Colleagues' Support

Participants emphasized the importance of having people, including department leaders, around them who supported their work. One aspect of support that many participants highlighted was mentorship. “I think mentorship is a huge component… huge aspect of almost every aspect of my career as far as the direction that I wanted to go.” (1010) Participants also described the importance of having a community of practice.

I think that's where community becomes really important, even amongst those trying to lead these initiatives, and having a community of support because at times, it gets difficult, where you feel like a lot of the initiatives are being challenged. And I think having that community of support of other people that understand the importance of the work, that you can kind of commiserate with and get motivation and move forward again. (1018)

Participants emphasized the importance of alignment with department chairs or other institutional leaders' vision for DEI leaders to succeed.

So it's a bigger lift. And that's what I mean about you really, if you're going to do this and do it right, you need to have a leader who has a vision. And you have to be a leader who is willing to say you know what? We're doing this … but it can't be the task of one person. It has to really be a collective. And for that to happen, an institutional leadership buy‐in has to be there. And if it's not, nothing's happening. (1014)

### 3.4 Internalized Domain (Roots)

Categories within the internalized domain included the aspects within a leader that influence their effectiveness. Internalized categories include the DEI leader's personal commitment to the work, feelings of inclusion, imposter syndrome, and tokenism.

#### 3.4.1 Personal Commitment to DEI Work

Participants described feeling personally driven to this type of work. Participants became interested due to personal experiences as an underrepresented minority.

I identify as a person who's underrepresented in medicine. So I think that comes natural… it's always been a focus of mine and something I've been involved in in terms of leadership and mentorship and education, all those different avenues, I think it just naturally made sense to continue to find roles that supported that. (1022)

A common challenge they faced was a strong desire to go above and beyond, driven by their genuine care for the work.

#### 3.4.2 Feelings of Inclusion

Participants explained that belonging and having space to be their authentic selves in a department is a crucial part of DEI work because it helps them feel valued as a person and that their work matters.

And without an equitable and an inclusive environment, all your diversity does is it gives you nice numbers, but it increases the chance that you'll have unhappy people because they don't feel included, or they're not equitable practices. (1001)

#### 3.4.3 Imposter Syndrome

Imposter syndrome has been previously defined as self‐doubt of intellect, skills, or accomplishments [21]. Participants described not feeling qualified: “I was not convinced that I was the right person necessarily to do harder stuff. Because I had been the person who had sort of gotten us to here with help, with lots and lots of help… But my chair and other people who convinced me I should apply, so I did.” (1012) For one participant, she reflected on how common it is for women to feel self‐doubt.

[I] just focus on me and my strengths and… stop limiting myself. I think that women a lot of times have issues or problems with that, because women, in large, are never taught to venture outside their comfort zone. (1013)

#### 3.4.4 Tokenism

Tokenism, or the practice of making perfunctory or symbolic efforts to engage underrepresented groups [22], was a concern for participants. Participants described situations in which they felt like tokens because the system wasn't genuinely committed to change.

At my hospital and my department, it was that window dressing again that we were talking about. It was like, yes, we want DEI, but we don't really want to change the system. We don't want to sacrifice this thing that we think is good or change the thing that we think is good because now we got to add the lens of DEI. (1010).

Other participants acknowledged that they might be a token or the departmental representative of diversity, and they accepted that, choosing to reframe themselves as trailblazers. “There were aspects of my career, basically, when I was the only African American or person of color in my department, where definitely tokenism was present. But I don't feel like that now. I feel more of a trailblazer for somebody who's been the first.” (1013).

## 4 Discussion

In our prospective qualitative study, we developed a deeper understanding of DEI leaders' experiences in their leadership positions, including challenges to their effectiveness. We expanded the allegory of the Gardner's Tale to DEI work broadly and applied it to our data because it explores how the environment where the DEI leader works impacts DEI leader effectiveness (Figure 2). The adapted allegory includes components of racism but considers historical discrimination and marginalization more broadly. In the adapted allegory, the DEI leader is the sunflower, positioned at the center of the ecosystem. Several institutionalized factors, such as the sun and cloud, impact the effectiveness of DEI leaders. Personally mediated factors are represented as the soil where the plant is rooted, along with the sunflower's relationship with the bees and other flowers. Lastly, internalized factors, through the characteristics of the roots, represent the perceived influence of the DEI leader's effectiveness. This allegory helps us visualize how a DEI leader operates within a dynamic environment that is constantly changing and often beyond the sunflower's control. Ideally, these interconnected factors are balanced, enabling DEI leaders to thrive and be highly effective in their efforts.

In the following paragraphs, we make recommendations based on the modifiable factors identified in the interviews that affect DEI leaders' success thus providing a practical framework by which department chairs and DEI leaders can assess their local environment and create a structure that facilitates role effectiveness.

Our participants described the variability in institutionalized support ranging from their clinical department, health system, medical school, and state legislation. The variability in department support, as described through differences in administrative positioning and variability in chair buy‐in through protected time, was a category that intersected with the minority tax category. This aligns with previous studies that have found that the minority tax contributes to disparities in leadership opportunities and promotion [12, 23, 24, 25, 26, 27]. Additionally, previous literature has linked minority tax with racism and discrimination, which contribute to feelings of isolation, marginalization, lower job satisfaction, and higher rates of turnover [28, 29]. This highlights a potential connection between institutionalized categories and internalized categories. We recommend DEI leaders and administration thoughtfully discuss how departmental resources and partnership with institutional leaders can be appropriately leveraged to support the goals of the DEI leadership role.

Many components of the personally mediated category are not unique to DEI positions. In fact, a recent literature review for DEI leaders found that key attributes to leadership generally include motivating others, fostering potential, inspiring trust, thinking strategically, setting goals and expectations, giving feedback, and being authentic [30]. Our study affirms many of these attributes and suggests that the DEI leaders not only require these common communication and leadership skills but also additional unique skills related to the sensitivities of the work and managing the personal impact this work has on their own well‐being. DEI leaders in this study identified the benefits of DEI and health equity‐specific leadership development programs, such as the Executive Leadership in Academic Medicine for Women Program and the Healthcare Executive Diversity and Inclusion Certificate offered by the AAMC. However, due to the current political challenges to DEI, several of these programs have been redesigned, paused, or canceled. In the current climate, DEI leaders may need to seek more general leadership opportunities, such as SAEM's Emerging Leader Development Program and Chair Development Program. This aligns with a commentary's recommendations on addressing minority tax by aligning career goals with institutional mission, using multiple mentors for career guidance, and negotiating supported time for scholarship [31].

Internalized factors specifically highlight the need for psychosocial support for our workforce. As we have shown previously, DEI work is often performed by those who are historically minoritized in medicine and face additional burdens navigating the culture of academic medicine. Several proposed interventions include developing culturally aware mentorship models and communities of practice [32]. A recent systematic review on mentorship for underrepresented in medicine physicians found four key themes to successful careers: (1) institutional alignment of programming, (2) tailored mentorship specific to local institutional needs, (3) the challenges of lack of racial and ethnic diversity and need for allies beyond concordance, and (4) the need for formal mentor training [33]. Individuals performing this work are often the only individuals in their department leading such efforts. School‐wide interdepartmental communities of practice, or nationwide specialty or other shared interest groups, can be a powerful source of motivation and mentorship for DEI leaders.

While our work focused on the experience of EM DEI leaders, we found similarities between EM DEI leadership challenges and those in the literature for other academic medicine DEI positions. A study of institutional DEI leaders, of which 53% held a dean position, found similar challenges in variability in role responsibility, mismatched institutional alignment, lack of evidence‐based practices, and personally exhausting work [9]. Unique to our study, we structure these challenges within an ecosystem framework that allows readers to consider the institutionally, personally, and internally mediated factors that lead to these experiences. The uniqueness of the ecosystem of an academic department, compared to a school or health system, likely poses different challenges. For example, from the perspective of a department of anesthesiology, one study proposes a leadership framework comprising senior leaders, a diversity and inclusion team, diversity and inclusion champions, and general membership [34]. Emergency medicine departments could use this structure as a starting place to support the DEI leader's role; however, additional research is needed to identify the optimal leadership structure for DEI leaders in emergency medicine.

### 4.1 Limitations

Findings of our work should be interpreted within the context of our interviews. All interviews took place in mid‐2024 and just before a national election in the United States. The findings reflect the sentiments of DEI leaders at a specific socio‐political time when there was high variability in state‐level DEI policies and a generally permissive federal policy. New guidance on the legal interpretation of longstanding civil rights laws has called into question which practices may remain. However, we believe that regardless of the political environment, there will remain an unmet need to ensure that all individuals have an opportunity to thrive, are free from discrimination, and have a strong sense of inclusion and belonging in their workplaces. Further, the fundamental concept of medicine to promote healthy communities for all remains an aspirational idea that has not yet been met. While our study focused on the experience of DEI leaders and their ecosystem, other individuals in the ecosystem were not captured. The recruitment strategy was an opt‐in system, and it is possible that participants who had particularly memorable (good or bad) DEI leadership experiences were more willing to participate. Likewise, our recruitment strategy used national DEI listservs, potentially resulting in overrepresentation of nationally engaged DEI leaders and more limited representation of less resourced DEI programs. This could have resulted in us characterizing a group of DEI leaders who have more resources than some of their peers. Additionally, there was likely variance in how our interview question stems were understood. Finally, this work was descriptive and exploratory, and we did not seek negative cases of the findings.

## 5 Conclusions

This study, in combination with prior research, has highlighted factors that affect the effectiveness of DEI leaders in emergency medicine. To develop effective DEI leaders, departmental leaders should consider how components within each aspect of their ecosystem support or hinder DEI leader success. Future directions will further explore how DEI leadership maps to more traditional leadership frameworks, including sustainability and succession planning. Finally, exploring emerging categories in a rapidly changing landscape may reveal new challenges and opportunities that define DEI leadership resilience.

## Author Contributions

R.E.T. contributed to the study concept and design, acquisition of data, analysis and interpretation of the data, drafting of the manuscript, critical revision of the manuscript for important intellectual content, and acquisition of funding. R.J.S. contributed to the analysis and interpretation of the data, drafting of the manuscript, and critical revision of the manuscript for important intellectual content. D.B. and A.I.P. contributed to the study concept and design, analysis and interpretation of the data, critical revision of the manuscript for important intellectual content, and acquisition of funding.

## Funding

This work is supported by the SAEMF/ADIEM Research Grant (AG2020‐0000000151) and the BerbeeWalsh Department of Emergency Medicine Research Pilot Award.

## Conflicts of Interest

The authors declare no conflicts of interest.

## Supporting information


**Appendix S1:** acem70322‐sup‐0001‐AppendixS1.zip.

## Data Availability

The data that support the findings of this study are available on request from the corresponding author. The data are not publicly available due to privacy or ethical restrictions.
