# Supplementary material for: Voices From Diversity, Equity, and Inclusion Leaders in Emergency Medicine, Understanding Their Experiences
Source: Acad Emerg Med. 2026 May 14;33:e70322. doi: 10.1111/acem.70322 (PMC13174904; doi:10.1111/acem.70322)
Supplement: Supplementary file 1 — Appendix S1: acem70322‐sup‐0001‐AppendixS1.zip. [file ACEM-33-0-s001.zip › acem70322-sup-0001-Supinfo1@Methods_Supplement_Codebook.pdf]

DEI Leader Codebook

Considerations in DEI Work	
Belonging and Inclusion (added 2-20-2025)	Participants describe wanting to feel like they belong or have community or the importance of building community.
Breadth of DEI	Participants describe the wide scope of DEI responsibilities, competing priorities, or the challenge of addressing many DEI domains simultaneously (e.g., race, gender, disability, equity, inclusion), often resulting in difficulty prioritizing or feeling spread too thin.
Buy In	Participants describe needing to build consensus or involving multiple people so that people accept what is built (i.e. a policy, etc.). Would also include people not being as supportive of the work as they had thought. Would include what generates credibility. Would also include when they are talking about engagement in DEI.
Censorship	Participants describe being limited, silenced, or constrained in what they can say, name, or pursue related to DEI work, including self-censorship due to fear of repercussions, legal concerns, or institutional pressure.
Disenfranchised	Person feels like the DEI work they do, doesn't or no longer matters. A feeling in a group or a person of having no power or opportunity. Deprived of the full opportunity to participate in community.
Imposter Syndrome	Self-doubt of intellect, skills, or accomplishments
Inaugural Role	Participants describe stepping into a newly created, first-time, or undefined formal role related to DEI work, often involving role ambiguity, lack of precedent, or building responsibilities from the ground up. This code includes moving from informal to formal title/role.
Interpersonal Skills	Includes conflict management, working in teams, negotiation, working with different personalities and people.
Leadership Experience	Not having experience running team, oversight, structure. Could also be the positive side of this, I know how to run teams, I know how to put in structure. Would also include characteristics that make a good leader
Long or Complex Coordination	Participants describe DEI work that requires extended timelines, coordination across multiple stakeholders, departments, or systems, and navigation of complex bureaucratic or organizational processes.
Minority Tax	The tax of extra responsibilities placed on minority faculty to others in the name of efforts to achieve diversity and the burden to be equivalent to others or lead burdensome efforts to secure accommodations.
Multiple Hats (Added 1-10-25)	Not saying people don't know how to prioritize (that would be breadth of DEI). When participants describe how the multiple roles they have gives them flexibility to do DEI work. Would also include people who have multiple roles in the institution and how that can help DEI work. Not simply mentioning that they have multiple roles, but talking about how multiple roles helps them (does not have to be specific to DEI roles)
Navigating the System	Participant talks about not knowing who to go to or learning about the structure of how things work
Pendulum Swing or New Headwinds	Participants describe shifts in institutional, political, or cultural climates that change support for DEI work over time, including cycles of progress and backlash or emerging barriers that slow or reverse momentum.
Pragmatism or Compromise	Participants describe adjusting goals, expectations, or strategies to align with practical constraints (e.g., institutional limits, resources, political climate),

	including making compromises or incremental changes when ideal or aspirational DEI goals are not feasible in the current context.
Professional Identity	Participants describe how their various identities influence how they approach DEI work. It can be emergency medicine identity or other things people attribute to their professional role.
Reporting Structure	Participants describe who they report to. Sometimes this includes complexities with reporting to multiple people.
Stereotype Threat	Participants describe awareness or fear that they may be judged, treated, or evaluated based on stereotypes about their identity, which affects their confidence, behaviour, or participation in DEI or professional spaces.
Strategic Planning	Participants describe intentional, forward-looking planning related to DEI work, including setting priorities, defining goals, sequencing activities, allocating resources, and developing formal or informal plans to guide implementation over time. Includes prioritization. Formal or informal planning.
Succession Planning	Participants describe planning (or lack thereof) for leadership transition, continuity, or sustainability of DEI efforts when individuals leave roles or institutions.
Tokenism	The practice of making only a perfunctory or symbolic effort. The appearance of one thing and the reality being different. Needs to be more than just not having resources.
Understanding	Participants describe a lack of understanding among colleagues, leaders, or institutions about the purpose, value, or goals of DEI work, or the need to repeatedly explain or justify why the work matters.
Urgency	Participants describe urgency or lack or urgency to work on DEI initiatives. Would include when the participants describe needing a frequent space to discuss DEI work or attempts to keep DEI at the front of mind.
Workarounds	Comments about changing how DEI work was labeled or creative solutions to continue to do DEI work in an evolving landscape. Usually related to legal considerations. You're trying to survive and continue doing DEI work.
Passions and Motivations for Doing DEI Work	
Identity Based Calls to Action	Participants describe being motivated to engage in DEI work due to aspects of their own identity or lived experience, leading them to feel a responsibility or obligation to act.
• LGBTQIA+ Identity Based Calls to Action	Participants describe motivation for DEI work rooted in LGBTQIA+ identity, lived experiences, or advocacy for LGBTQIA+ communities.
• Race or Ethnicity Identity Based Calls to Action	Participants describe motivation for DEI work rooted in racial or ethnic identity, experiences of racism, or advocacy for racially or ethnically marginalized groups.
• Sex or Gender Identity Based Calls to Action	Participants describe motivation for DEI work rooted in sex or gender identity, experiences of sexism or gender-based inequities, or advocacy for gender equity.
Impact	Positive feedback from peers or department about impact or feeling like you are making a difference. This code is bigger than just getting started in DEI. Would including wanting to give back.
Inspired by Others Work in Area	Participants describe being motivated by mentors, peers, role models, or visible DEI leaders whose work influenced or inspired their own involvement.
Personal Experiences	Feeling personally driven to do DEI work.

Service (added 1-17-2025)	Doing unpaid work, funding projects out of your own pocket, and doing the work before your recognized for it. Can lead to future more official titles or resources but not always. Would include doing the work because you have a passion for it. Mentoring and committee work are often considered service activities.
Politics	
Current Events	Participant describe how current events motivated or impacted their DEI work. Should be used when Federal or state politics doesn't apply.
Federal or National Politics	Participants describe how federal laws, national policies, court rulings, or national political discourse affect DEI initiatives, constraints, or strategies.
Pushback	Participants describe situations where the received pushback or resistance to their DEI work. More than just general challenges to DEI work but needs to be explicitly things done against DEI (eg. white man's group, twitter attack)
State Politics	Participants describe how state-level laws, regulations, or political actions influence DEI work, including restrictions, funding changes, or mandates.
Professional Development	
Lifelong Learning	Participants describe ongoing, self-directed learning related to DEI, including reading, reflection, training, or staying current with evolving concepts and best practices.
Meeting Criteria for Promotion	Discussions surrounding whether DEI skills, experiences, and performance facilitated formal professional advancement.
Mentorship	Should only be situations where the participant is receiving mentoring or peer mentoring.
On the Job Training	Participants describe learning on the job or not having a roadmap or a description of what they should do. Solving problems as they go. Experiential Learning
Training Programs	Programs specific to being a leader or DEI leader. Not a degree based program.
Local Training Programs	Participants describe institution-based, regional, or local training opportunities related to leadership or DEI development.
National Training Programs	Participants describe nationally recognized programs, fellowships, or leadership initiatives focused on DEI or leadership development.
Relationships	
Chair	Participants describe their relationship with their department chair, including support, oversight, advocacy, or barriers related to DEI work.
Transition Between Chairs	Participants describe changes, disruptions, or opportunities related to DEI work that occur during leadership turnover at the chair level.
Community of Practice	Participants describe informal or formal groups of peers engaged in shared DEI learning, problem-solving, or mutual support.
Colleagues or Support Staff	Core leadership piece, building a team the things you need for that.
Other Department Leaders	Participants describe interactions with leaders in other departments related to collaboration, support, or resistance to DEI initiatives.
Other Institutional Leaders	Participants describe interactions with institutional leadership beyond the department (e.g., deans, provosts, system leaders) that influence DEI work.
Words vs. Action	Participants describe scenarios where there are alignment or nonalignment between words and actions. Can be positive and negative and could be their Chair, the Dean, the Provost or others.

DEI Leader Codebook

**Resources**	
Administrative Support	Participants describe logistical, staffing, or operational support that facilitates (or is missing from) DEI efforts.
Budget (institution and-or Department)	Participants describe financial resources, funding constraints, or budgetary decision-making related to DEI work.
Protected Time	Participants describe having—or lacking—formally allocated, compensated time to engage in DEI work as part of their role. Includes Financial resources or instances where they are talking about not getting paid for work
**Work Performed**	
Administrative Positioning	The DEI leader's role within their department, including specific leadership responsibilities, decision-making authority, direct communication channels to the Chair, and access to financial resources
Collaboration across Other Sectors	Participants describe cross-unit or cross-role collaboration within their department to advance DEI initiatives.
Community Engagement	Participants describe DEI work involving partnership or engagement with communities outside the institution.
Education or Didactics	Participants describe teaching, training, presentations, or curriculum development related to DEI.
Generating and Tracking DEI Outcomes	Participants describe collecting data, measuring impact, or evaluating outcomes of DEI initiatives.
Health Equity	Participants describe clinical or systems-level work aimed at reducing healthcare disparities or improving equitable health outcomes.
Microaggression based	Participants describe DEI work related to addressing, responding to, or preventing microaggressions in clinical, educational, or professional settings.
Pathways to Medicine or Emergency Medicine	Would include mentoring students or other learners.
Recruitment	Participants describe efforts to recruit diverse faculty, staff, trainees, or learners.
Wellness and Retention	Participants describe DEI work focused on supporting well-being, reducing burnout, and retaining marginalized or underrepresented individuals.
