# Supplementary material for: Voices From Diversity, Equity, and Inclusion Leaders in Emergency Medicine, Understanding Their Experiences
Source: Acad Emerg Med. 2026 May 14;33:e70322. doi: 10.1111/acem.70322 (PMC13174904; doi:10.1111/acem.70322)
Supplement: Supplementary file 1 — Appendix S1: acem70322‐sup‐0001‐AppendixS1.zip. [file ACEM-33-0-s001.zip › acem70322-sup-0002-Supinfo2@Methods_Supplement_Interview_Guide.pdf]

Semi-Structured Interview Guide

General:

1. Can you tell me about your title? How did you come into your position? When did you realize this type of work was what you wanted to do?
    a. What do you see core responsibilities of your role?
    b. Overall, has being a DEI leader been a positive or negative experience? How so?

Opportunities:

1. Take me back to when you started your position. What drew you into it?
    a. What external forces led to your involvement?
    b. Were there internal drivers?
2. Tell me about an achievement you had in your position as a DEI leader.
    a. What made it particularly significant?
    b. What factors made the achievement possible?
    c. Have you ever experienced an unexpected ally? Who was it? What made it unexpected? What made the relationship valuable?
        i. *Probe: If not discussed already, ask:* What has your relationship with your chair been like?
    d. How have you used the available resources to enact change?
    e. Walk me through the impact of funding on the critical components of your role
    f. How does the department value your work – through time, funded effort, or salary differentials?
3. Tell me about a time when you used specific leadership skills to be effective.
    a. How does one go about getting those?
    b. What formal or informal processes helped you get there?
    c. How have you found your mentors?
    d. How have you created a community of practice?

Barriers:

1. Tell me a time you hoped to enact change, but it didn't come to fruition, *OR* Can you recall a time when you wanted to enact change but couldn't? Tell me about it.
    a. What did you learn from that experience? What change needed to happen to have a more positive outcome?
2. In the current DEI climate, can you describe an instance in which your work has changed?
    a. Tell me about internal/external forces.
    b. Belonging vs social isolation
    c. How have current laws, such as the SCOTUS/SFFA ruling, or proposed legislation, or the political landscape impacted your work?
    d. How have current events, such as the conflict in Gaza, impacted your work?
3. Share with me how your work interfaces with HR?
4. How does DEI work relate to academic freedom of expression?
5. How do you manage your passion for DEI versus what is practical?\

Promotion:

1. What conversations have you had about promotion?
    a. *Probe:* Can you tell me about a conversation in your Chair/department leader has valued diversity?

Vision:

1. Tell me 2-3 things that motivate you about your position?
2. Tell me 2-3 things that you find most discouraging?
3. Suppose that you had 5 minutes with a mentee interested in becoming a DEI leader. What would your advice be?

Is there anything else we haven't talked about yet?

Demographics:
Can you tell me about the title/role you have as a DEI leader in Emergency Medicine?
Can you tell me about how you identify? This could include things like your age, gender, race, ethnicity, sexual orientation, religion, and whether you have a disability.

Probing Questions:

- Can you tell me more about ..?
- Is that something that most people do?
- What does that mean for you?
- What does that mean for the patient?
- So what I understand is that……. Is that correct?
- And then what..?
- What happens?
- Repeat what was just said
- What do you think about that?
- What needs to be done about that?
- Can you describe that to me a little more?
- Can you explain ..?
- What do they do..? What did you do?
- Is that important? Why is that important?
- What else?
- Is there something else you would like to bring up about that?
