# Supplementary material for: Voices From Diversity, Equity, and Inclusion Leaders in Emergency Medicine, Understanding Their Experiences
Source: Acad Emerg Med. 2026 May 14;33:e70322. doi: 10.1111/acem.70322 (PMC13174904; doi:10.1111/acem.70322)
Supplement: Supplementary file 1 — Appendix S1: acem70322‐sup‐0001‐AppendixS1.zip. [file ACEM-33-0-s001.zip › acem70322-sup-0003-Supinfo3@Supplementary_Index.docx]

**Methods_Supplement_Interview_Guide.pdf**

Semi-structured interview guide developed and implemented for the study (PDF, 2 pages, 105 KB).

**Methods_Supplement_Codebook.pdf**

Complete codebook utilized for all DEI leaders. Note that some codes were later adapted, modified, or merged to align with the Gardner’s Tale Framework (PDF, 4 pages, 147 KB).
